# Advanced High-Content Phenotypic Screening to Identify Drugs That Ameliorate the Inhibition of Skeletal Muscle Cell Differentiation Induced by Cancer Cachexia Serum

**DOI:** 10.3390/ph18040445

**Published:** 2025-03-21

**Authors:** Atsushi Nakane, Hiroyuki Nakagawa, Hidetaka Nagata

**Affiliations:** Sumitomo Pharma Co., Ltd. 1–98, Kasugade-naka 3-chome, Konohana-ku, Osaka 554-0022, Japan; atsushi.nakane@racthera.co.jp (A.N.); nakagawah2@sc.sumitomo-chem.co.jp (H.N.)

**Keywords:** cancer cachexia, muscle differentiation, high-content phenotypic screening, HDAC inhibitors

## Abstract

**Background/Objectives**: Cancer cachexia (CC) is a prevalent and debilitating syndrome in cancer patients, characterized by severe muscle and weight loss, leading to increased mortality and reduced quality of life. Despite the significant impact, effective treatments are lacking due to an incomplete understanding of its underlying mechanisms. In this study, we aim to develop drugs that ameliorate the inhibition of muscle differentiation induced by CC. We established an advanced, high-content phenotypic screening system using the serum of cancer patients and identified potential compounds. **Methods**: We used cancer patients’ sera as pathophysiological stimuli in our screening system to evaluate their effects on muscle atrophy and differentiation. Various histone deacetylase (HDAC) inhibitors were tested for their efficacy. The system’s translational relevance was validated by comparing results with clinical data and in vivo cachexia models. **Results**: Using our screening system, we evaluated several cancer patients’ sera and found that they reflect clinical features of cancer cachexia. In addition, HDAC inhibitors, particularly those with broad-spectrum inhibition, showed promise as agents to ameliorate the inhibition of muscle differentiation induced by CC sera. This system’s findings were consistent with clinical and in vivo data, highlighting its potential for identifying new drugs. **Conclusions**: The high-content phenotypic screening system effectively mimics some key aspects of CC pathophysiology on skeletal muscle, providing a valuable tool for drug discovery and understanding CC mechanisms. The translational relevance of our system offers a promising avenue for therapeutic advancements in the management of cancer cachexia, with the potential to improve patient outcomes and quality of life.

## 1. Introduction

Cancer cachexia (CC) is a complex catabolic syndrome characterized by the ongoing, involuntary loss of muscle mass and body weight, with or without the loss of adipose tissue. This condition cannot be reversed by nutritional supplementation and leads to progressive functional impairment [1]. CC is a multi-organ syndrome, leading to systemic metabolic changes and systemic inflammation. However, the mechanisms cause impairment in these organs have not been studied in depth [2]. Overall, CC affects about 80% of cancer patients and leads to death in 20–30% of cases [3]. The prevalence of this comorbidity varies by cancer type and stage, reaching up to 70% in pancreatic cancer, 60% in gastroesophageal and head-and-neck cancers, 40–50% in lung, colorectal, and certain hematological cancers, and 15–25% in breast and prostate cancers [4]. The complexity and heterogeneity of this syndrome are due to the involvement of various mediators (such as systemic pro- and anti-inflammatory mediators, hormones, neuropeptides, and tumor-derived factors) and signaling pathways, making clinical management very challenging [5]. Despite imposing a significant burden on the quality of life of cancer patients, scientific knowledge of the condition remains limited. The underlying pathophysiological mechanisms of this disorder are not fully understood; therefore, there are no approved standard treatments for CC. In two randomized controlled trials involving patients with advanced CC, the TNFα receptor–blocker etanercept [6], and the TNFα specific monoclonal antibody infliximab [7], did not prevent muscle atrophy. However, many investigational drugs have shown promise in clinical and preclinical studies. For instance, anti-Growth Differentiation Factor 15 (GDF-15) [8] and the histone deacetylase inhibitor (HDACi) AR-42 [9] have shown efficacy in murine CC models. Taken together, continued research into novel therapeutic targets and mechanisms is still essential to investigate the interplay of dysregulated mechanisms [10].

The most detrimental aspect of CC is muscle loss due to atrophy. Skeletal muscle mass is maintained through a delicate balance between protein anabolism and catabolism [11]. During tumor progression, often accompanied by CC, this balance is disrupted [12], driven by the secretion of various pro-inflammatory catabolic factors by immune and cancer cells, such as tumor necrosis factor-α (TNFα) [13]. Another aspect that may potentially contribute to the loss of skeletal muscle in CC is impaired skeletal muscle regeneration. Skeletal muscle has a high capacity for self-regeneration in response to injury [14]. If skeletal muscle is assumed to be injured by CC, it is hypothesized that it also undergoes regeneration, but this process is impaired, contributing to muscle loss. Several studies have noted a correlation between inflammation and cachexia, with inflammation being a significant factor in weight loss among cancer patients [15,16]. Inflammatory cytokines, including TNFα and TWEAK, reduce the expression levels of key muscle differentiation factors such as troponin T type 1 (*TNNT*), myogenic differentiation factor 1 (*MyoD*), myocyte enhancer factor 2C (*MEF2C*), and myogenin (*MYOG*) [17,18,19,20].

Most mechanistic analyses of CC have been conducted using animal models. However, findings from existing preclinical models have not been consistently replicated in studies using human muscle biopsy samples or in successful clinical trials or drug discovery [5]. Differences in results between human and animal model studies could be partly due to physiological differences between species, tumor biology, tumor host interactions, pre-existent co-morbidities, previous treatments for cancer, or lack of interaction between the tumor and the host immune system [5,21].

To establish a cachexia-induced phenotype, various methods and agents have been used to induce skeletal muscle loss. These include direct agents such as cell medium from tumor cells [22], inflammatory factors [13], hormones [23], and chemotherapeutic drugs [24]. There are also reports of using ascites from cancer patients to stimulate the cachexia phenotype [25]. However, no in vitro evaluation system has been established that fully recapitulates the complexity of cachexia as a systemic inflammatory condition driven by multiple inflammatory mediators.

We have developed a novel, human, high-content phenotypic screening system that mimics some key aspects of the pathophysiology of CC on skeletal muscle. To mimic the systemic conditions, we used cancer patient sera as pathological stimuli. We evaluated the effects of patient serum on muscle atrophy and muscle differentiation, a key step in muscle regeneration, and found a substantial inhibition of muscle differentiation. Using this screening system, we evaluated various types of sera from cancer patients to identify remarkable effects on the trajectory of CC. Additionally, we conducted chemical screening using commercially available chemical libraries and identified HDAC inhibitors as active compounds. Various HDAC inhibitors were evaluated, and their target profiles were analyzed. Furthermore, our highly translational human phenotypic screening system that mimics the complex effects of cancer cachexia on skeletal muscle was validated, contributing to drug discovery and a clearer understanding of the mechanisms of cachexia.

## 2. Results

### 2.1. Effects of Cancer Patient Serum on Human Skeletal Muscle Differentiation and Atrophy In Vitro

To develop an in vitro phenotypic screening system to study the effects of cancer cachexia (CC) on skeletal muscle, we utilized human cancer patient serum as a pathophysiological stimulus and commercially available human skeletal muscle myoblasts (HSMMs) as a human cell system [26].

**Figure 1:**
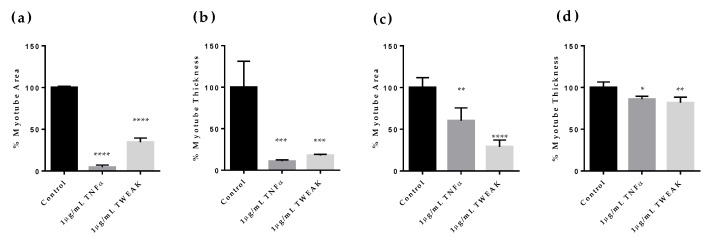
Effects of TNFα or TWEAK on myotube area and thickness at different times. Quantification of myotube area and myotube thickness caused by TNFα or TWEAK. (**a**) Percentage of myotube area on Day 4 after TNFα or TWEAK treatment from Days 0 to 4. (**b**) Percentage of myotube thickness on Day 4 after TNFα or TWEAK treatment from Days 0 to 4. (**c**) Percentage of myotube area on Day 9 after cytokine treatment from Days 5 to 9. (**d**) Percentage of myotube thickness on Day 9 after cytokine treatment from Days 5 to 9. Percentage of myotube area per field and percentage of myotube thickness per field are quantified by normalizing to 0% for cells cultured in expansion medium as undifferentiated cells and 100% for cells cultured in differentiation medium containing human normal serum. All values are means ± standard deviations (*n* = 4). * Denotes a significant difference from control at *p* < 0.05, ** at *p* < 0.01, *** at *p* < 0.001, and **** at *p* < 0.0001 (Dunnett’s multiple comparisons test).

First, to validate the effects of the stimulus on skeletal muscle, we utilized the cytokines TWEAK and TNFα, which have been previously reported to induce muscle atrophy and differentiation inhibition [27,28,29]. Myotubes were differentiated in HSMMs with the simultaneous addition of TNFα or TWEAK and cultured for four days. The myotube area and myotube thickness were evaluated on Day 4 (Figure 1a,b). Skeletal muscle mass was detected by immunostaining myotubes with myosin heavy chain (MHC) and quantifying myotube area and thickness using microscopy. Compared to the control (100%), TNFα and TWEAK significantly reduced the myotube area (TNFα by 4.7%, *p* < 0.0001; TWEAK by 34.7%, *p* < 0.0001) and thickness (TNFα by 10.8%, *p* < 0.001; TWEAK by 18.0%, *p* < 0.0001) on Day 4. Next, after the induction of differentiation in myotubes, TNFα or TWEAK was added, starting on Day 5. The effects of TNFα or TWEAK on myotubes was assessed by measuring the myotube area and thickness on Day 9 (Figure 1c,d). On Day 9, TNFα and TWEAK significantly reduced the myotube area (TNFα by 60.2%, *p* < 0.001; TWEAK by 29.0%, *p* < 0.0001) and thickness (TNFα by 85.9%, *p* < 0.05; TWEAK by 81.7%, *p* < 0.01), confirming the inhibitory effects of TNFα and TWEAK on muscle differentiation and the induction of muscle atrophy. These results indicate that the effects of TNFα and TWEAK not only induce atrophy but also inhibit differentiation by altering the timing of stimulation.

**Figure 2:**
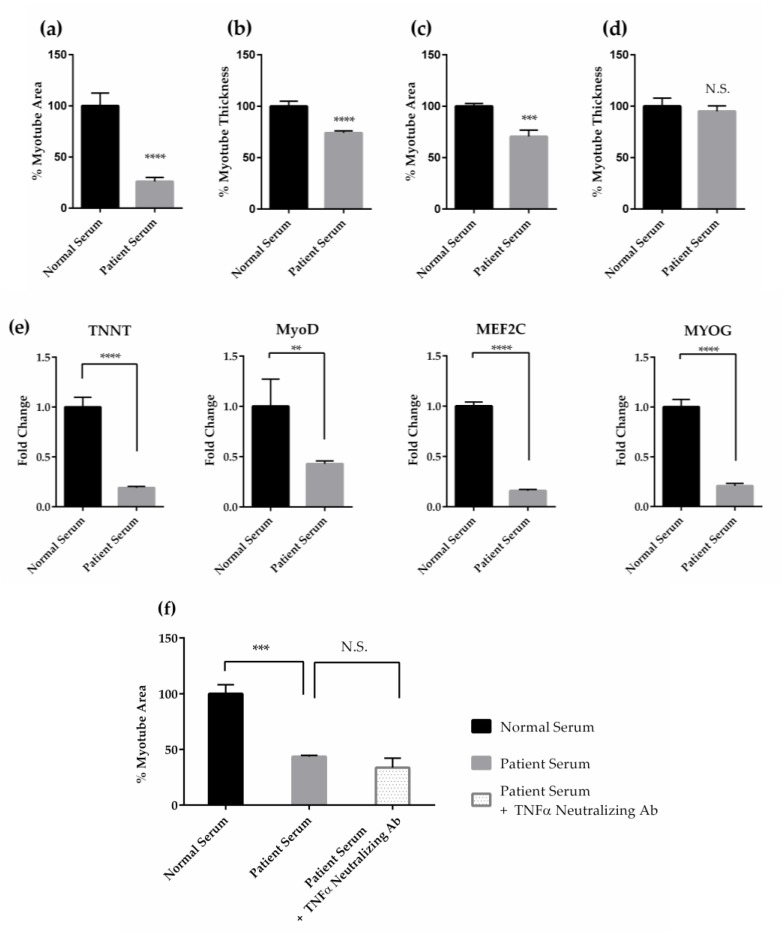
Effects of cancer patient serum on myotubes and their myogenesis-related gene expression. Quantification of % myotube area (**a**) and % myotube thickness (**b**) on Day 4 after cancer patient serum E (described in Table 1) treatment from Day 0 to Day 4. Quantification of % of myotube area (**c**) and % of myotube thickness (**d**) on Day 9 after cancer patient serum E treatment from Days 5 to 9. Percentage of myotube area per field and % of myotube thickness per field are quantified by normalizing to 0% for cells cultured in expansion medium as undifferentiated cells and 100% for cells cultured in differentiation medium containing human normal serum. (**e**) Effects of serum on the expression level of representative myogenesis-related genes. From the left, the expression level of *TNNT*, *MyoD*, *MEF2C*, and *MYOG* on Day 4 after cancer patient serum E treatment from Days 0 to 4. All values represent mean ± standard deviation (*n* = 4) of fold change in mRNA expression, normalized to *B2M* using the ∆∆Ct method. (**f**) Quantification of % of myotube area decrease caused by cancer patient serum E and the effects of TNFα using a neutralization antibody. Percentage of myotube area per field is quantified by normalizing to 0% for cells cultured in expansion medium as undifferentiated cells and 100% for cells cultured in differentiation medium containing human normal serum. All values are means ± standard deviations (*n* = 3). ** Denotes a significant difference from control at *p* < 0.01, *** at *p* < 0.001, and **** at *p* < 0.0001 (Dunnett’s multiple comparisons test for (**f**) or Student T test for (**a**–**e**). Abbreviations in the figure: N.S., not significant. Ab, antibody.

Furthermore, we evaluated the effects of cancer patient serum on muscle differentiation using human serum from patients with grade III colon cancer (serum E in Table 1). A high incidence of cachexia in patients with grade III or higher cancer has been reported. These results showed that the myotube area (26.1%, *p* < 0.0001) and myotube thickness (74.0%, *p* < 0.0001) were significantly decreased after the addition of cancer patient serum for four days, compared to normal serum (Figure 2a,b). In contrast, when cancer patient serum was added to differentiated myotubes, and the myotube area and thickness were evaluated on Day 9, the myotube area showed a significant reduction (70.6%, *p* < 0.001) compared to the normal control, but the thickness showed a slight reduction with no significant change (95.9%) (Figure 2c, d). These results establish our in vitro screening system that can reproduce the muscle differentiation and atrophy observed clinically in CC.

To further confirm the inhibition of muscle differentiation by serum from cancer patients, we conducted a gene expression analysis of key muscle differentiation factors on Day 4 using RT-qPCR (Figure 2e). Beta-2 microglobulin (*B2M*) was used as an internal control. Adding cancer patient serum to the muscle differentiation evaluation system significantly reduced the expression levels of *TNNT*, *MyoD*, *MEF2C*, and *MYOG* compared to the control. Changes in expression levels compared to normal serum were 0.19-fold (*p* < 0.0001), 0.43-fold (*p* < 0.01), 0.16-fold (*p* < 0.0001), and 0.21-fold (*p* < 0.0001), respectively. These results support the inhibition of muscle differentiation in CC by significantly suppressing the expression of muscle differentiation-related genes.

To evaluate the contribution of TNFα to the inhibitory effects of cancer patient serum on muscle differentiation, we tested the effect of TNFα-neutralizing antibodies. The addition of TNFα-neutralizing antibodies did not reduce the inhibition of muscle differentiation induced by cancer patient serum (Figure 2f). These results suggest that multiple factors, not just TNFα, are present in CC serum and that various molecules in combination contribute to the inhibition of muscle differentiation.

### 2.2. Establishment of a High-Content Phenotypic Screening System Using Cancer Patient Serum for Drug Screening

**Figure 3:**
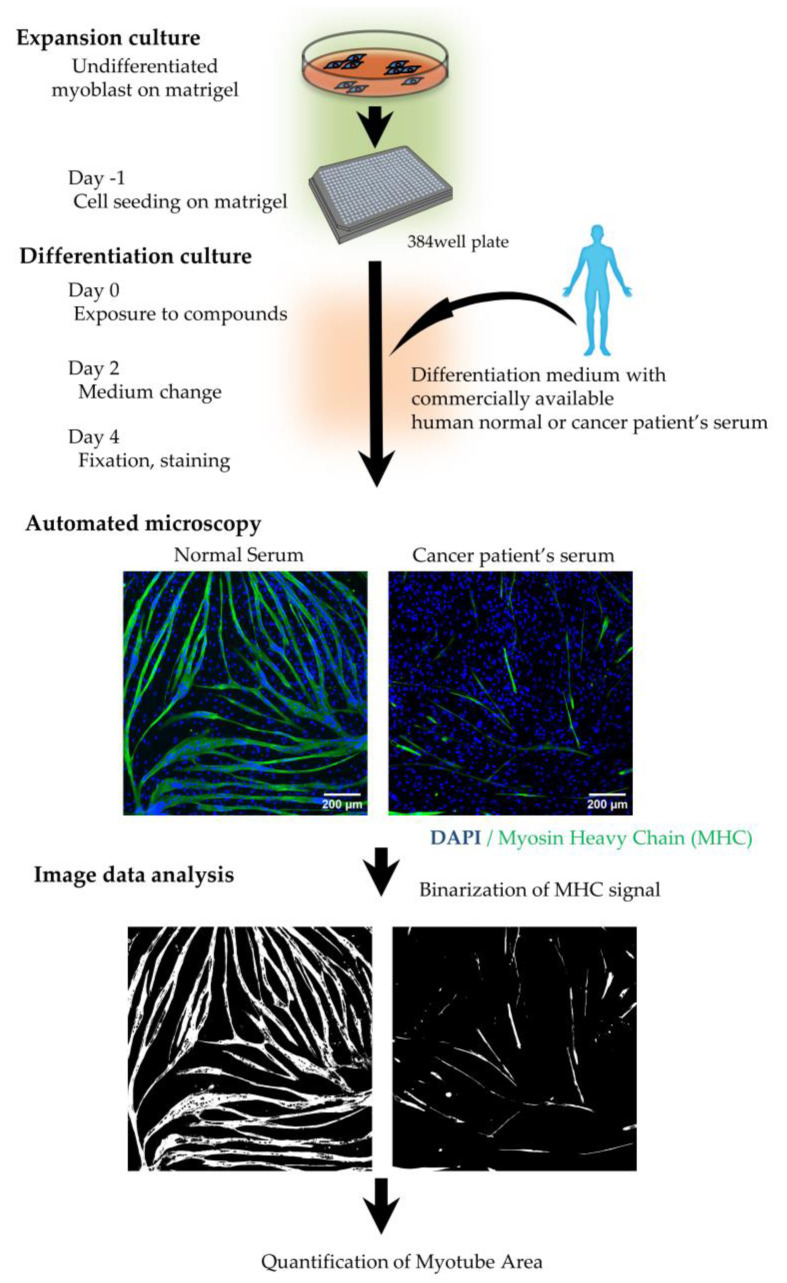
Schematic flow of high-content, phenotypic screening for the effect of cancer patient serum on myotubes. Schematic representation of the high-content, phenotypic screening flow and representative image (10× magnification) of myoblasts and myotubes on Day 4 using 2% human normal serum (left image) or 2% human serum from a cancer patient (right image). Nuclei are stained blue (DAPI), and green indicates the expression of fast skeletal myosin heavy chain. Scale bars are set to 200 μm.

We constructed and optimized a high-content phenotypic screening system to evaluate various cancer patient sera and to identify drugs that ameliorate the suppression of muscle differentiation caused by cancer cachexia. Figure 3 shows the schematic flow of our high-content phenotypic screening. This system uses a 384-well plate format (Figure 3). HSMMs were expanded and seeded into 384-well plates and differentiated for four days in the presence of normal or patient serum, stained with MHC, and imaged using an automated imaging system. Representative microscopy images show differentiation on Day 4 with normal serum (left) and cancer patient serum (right; patient serum E in Table 1). The myotube area was quantified from the acquired images. This high-content phenotypic screening system allows for the efficient screening of various patient sera for muscle differentiation inhibition and compounds that reduce this inhibition.

**Figure 4:**
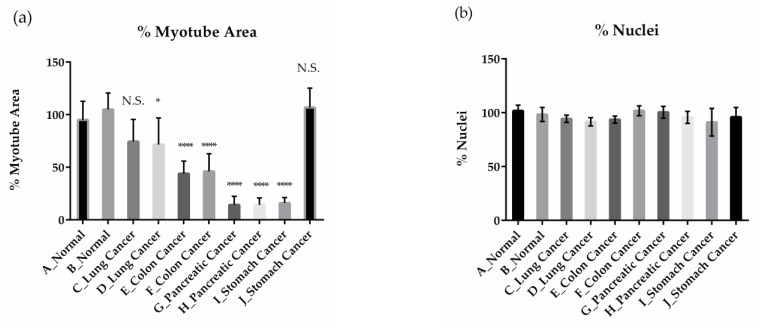
Effect of various types of cancer patient serum on myotube area and nucleus count. Quantification of % of myotube area per field (**a**) and % of nuclei per field (**b**) on Day 4 in differentiation medium containing normal sera (A or B) or cancer patient sera (C, D, E, F, G, H, I, or J). All values are means ± standard deviations (*n* = 4). Percentage of myotube area per field is quantified by normalizing to 0% for cells cultured in expansion medium as undifferentiated cells and 100% for cells cultured in differentiation medium containing normal human serum. Percentage of nuclei is quantified by normalizing to 0% for non-seeding well and 100% for cells cultured in differentiation medium containing normal human serum. * Denotes a significant difference from normal serum (data of serum A and B were combined into a single normal serum-treated group for statistical analysis) at *p* < 0.05, and **** denotes a significant difference at *p* < 0.0001 (Dunnett’s multiple comparisons test). Abbreviations in the figure are: N.S., not significant.

For optimization purposes, we first compared the effects of various types of cancer patient serum on the inhibition of muscle differentiation (Table 1). We tested two samples of normal human serum (serum A and B) and eleven samples of cancer patient serum (serum C, D, E, F, G, H, I, J, K, L, and M). Sera from lung cancer (serum D), colon cancer (serum E and F), pancreatic cancer (serum G, H, K, L, and M), and gastric cancer (serum I) patients significantly inhibited muscle differentiation compared to normal serum (Figure 4a, Figure S1). In contrast, sera from colon cancer (serum C) or gastric cancer (serum J) patients did not significantly inhibit muscle differentiation (Figure 4a). Cell count results showed no changes in cell number between normal and cancer patient sera, indicating that cancer patient sera inhibit muscle differentiation without affecting cell viability (Figure 4b). To verify screening system accuracy, we selected the optimal cancer patient serum based on the *Z’*-factor, a screening precision indicator. The *Z’*-factors were calculated by setting the condition of cancer patient serum treated with DMSO as the negative control and the condition of normal serum treated with DMSO as the positive control. The formula used for the calculation was as follows:

$$Z\;\prime =1-3\times \;\frac{S.D.positive+S.D.negative}{MEANpositive-MEANnegative}$$

Among these evaluated sera, serum I showed the highest *Z’*-factor (0.298). Bar et al. suggested that assays with a *Z’*-factor between 0 and 0.5 can detect useful compounds without generating excessive false positives if appropriate hit thresholds are set considering the value of S.D. _negative_/S.D. _positive_ [30]. Therefore, serum I was considered optimal as a pathophysiological stimulus for CC in compound screening.

### 2.3. Drug Screening Using High-Content Phenotypic Screening with Cancer Patient Sera

**Figure 5:**
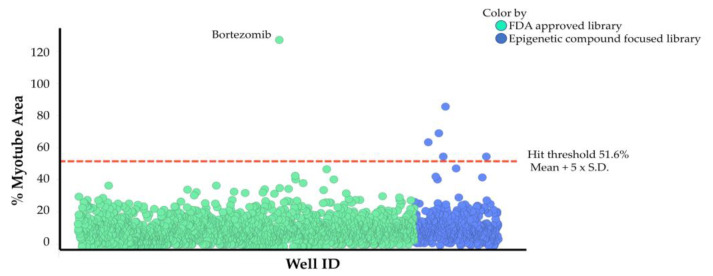
High-content phenotypic screening to reduce muscle differentiation inhibition caused by cancer patient serum. The effects of an FDA-approved drug library (712 compounds, green plots) and an epigenetic compound focused library (95 compounds, blue plots) at concentrations of 1 μM (*n* = 1) and 10 μM (*n* = 1) were assessed. Percentage of myotube area is quantified by normalizing to 0% for cells cultured in expansion medium as undifferentiated cells and 100% for cells cultured in differentiation medium containing normal human serum. Muscle differentiation inhibition was induced by cancer patient serum I. The hit threshold was set at the mean value of the DMSO control treated with patient serum I plus five times its standard deviation, resulting in a threshold of 51.6%. The dotted red line represents the hit threshold. Abbreviations in the figure are as follows: S.D., standard deviation. A dose-dependent evaluation was performed on various HDAC inhibitors based on identified compounds (0.0032, 0.016, 0.08, 0.4, 2, 10 μM) (Figure 6). The HDAC family consists of 11 subtypes, and the subtype selectivity of the compounds used in this study is summarized in Supplemental Table S1 based on information from previously reported papers [31,32,33,34,35,36,37]. The HDAC6-selective inhibitor CAY-10603 [34] showed the ability to reduce muscle differentiation inhibition, suggesting that HDAC6 contributes to muscle differentiation inhibition. In contrast, the HDAC8-selective inhibitor PCI34051 [34] did not show reducing effects. Class I HDAC-selective inhibitors (MS-275 [34], SB939 [31] and chidamide [32], TC-H 106 [35]) showed partial reducing effects on muscle differentiation. Pan-HDAC inhibitors (4-iodo SAHA [38], ITF2357 [32], KD5170 [33], TSA [33], AR-42 [37]) showed significant reducing effects, except for SAHA [32]. Interestingly, SAHA, which has a similar HDAC subtype inhibition profile to 4-iodo SAHA, only showed partial reducing effects.

Using our high-content phenotypic screening system with cancer patient serum I, we aimed to identify compounds that reduce the inhibition of muscle differentiation. We screened an FDA-approved drug library containing 712 drugs and an epigenetic compound focused library containing 95 compounds at concentrations of 1 μM and 10 μM to evaluate at multiple concentrations and avoid missing potential hits (Figure 5). All plates in this screening had *Z’*-factors above zero (0.08–0.60) and low S.D._negative_/S.D._positive_ values (0.03–0.16), meeting the data quality acceptance criteria for high-content screening [30]. The hit threshold was set at the mean value of the DMSO control treated with patient serum I plus five times its standard deviation, resulting in a threshold of 51.6%. Compounds that exceeded the hit threshold at either 1 µM or 10 µM were considered hits. The FDA-approved library screening identified the proteasome inhibitor bortezomib as a hit. The epigenetic compound focused library screening identified four HDAC inhibitors (MS-275, ITF2357, SB939, HC-toxin) and one BET inhibitor (PFI-1) as hits. However, PFI-1 did not show efficacy in subsequent reproducibility tests using different cancer patient sera. The screening identified several promising active compounds, with HDAC inhibitors showing the highest potential to reduce the inhibition of muscle differentiation by cancer patient serum.

### 2.4. The Effects of AR-42 on Muscle Differentiation Inhibition by Cancer Patient Sera

**Figure 6:**
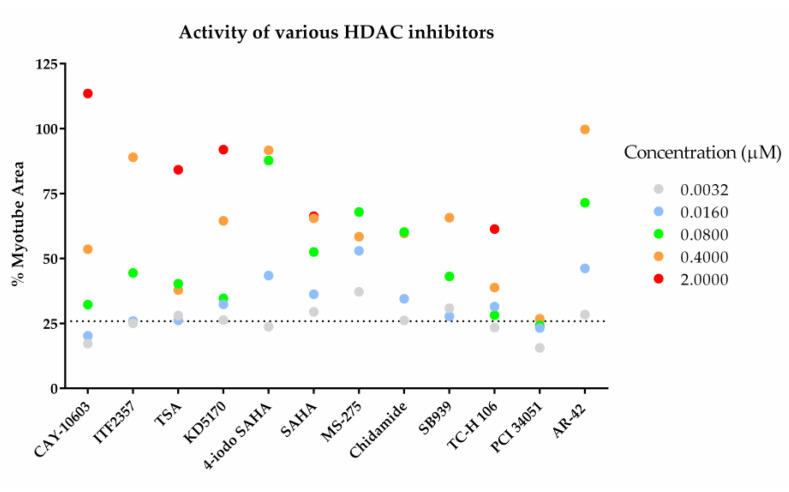
Dose response analysis of various profiled HDAC inhibitors. Effects on myoblast differentiation were evaluated in the presence of twelve different HDAC inhibitors. Percentage of myotube area was quantified by normalizing to 0% for cells cultured in expansion medium as undifferentiated cells and 100% for cells cultured in differentiation medium containing human normal serum as differentiated cells. Muscle differentiation inhibition was induced by cancer patient serum M. All values are means (*n* = 2). The dotted line represents the value seen with the DMSO control. The concentrations of the evaluated compounds were 0.0032, 0.016, 0.08, 0.4, 2, and 10 µM, and the plots are color-coded according to concentration. At the highest concentration of 10 µM, the activity of all evaluated compounds was lower than the maximum activity value, so it was not represented in this figure.

**Figure 7:**
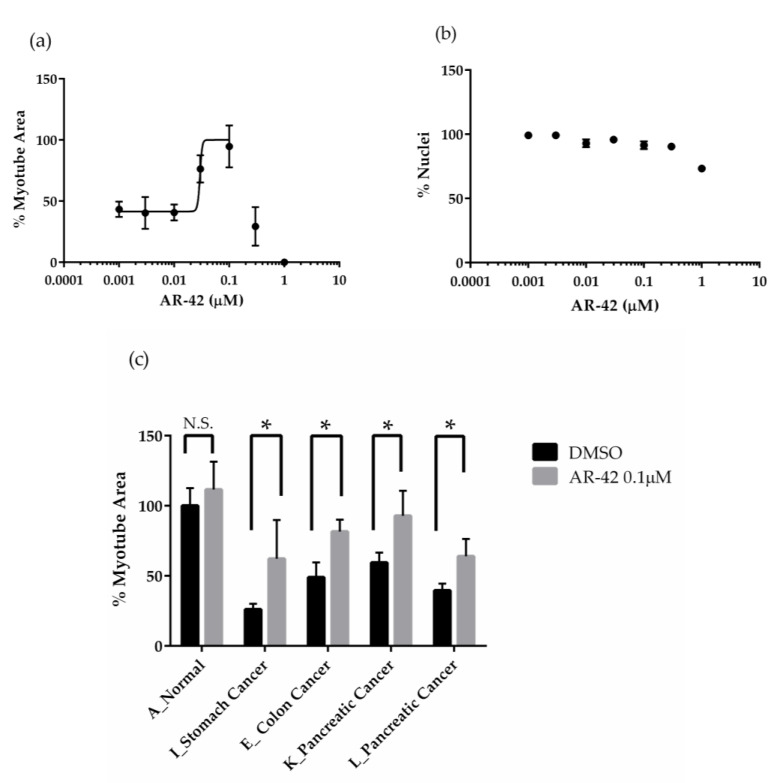
Effect of AR-42, a pan-HDAC inhibitor, on muscle differentiation inhibition induced by various cancer patient sera. (**a**) Quantification of MHC-positive area per field and number of nuclei per field in various types of normal or cancer patient sera on Day 4 in the presence of DMSO or 0.001 to 1 μM of AR-42 using differentiation medium containing 2% normal serum (serum A) or cancer patient serum (serum E). Percentage of myotube area was quantified by normalizing to 0% for cells cultured in expansion medium as undifferentiated cells and 100% for cells cultured in differentiation medium containing human normal serum as differentiated cells. (**b**) Percentage of nuclei was quantified by normalizing to 0% for non-seeding well and 100% for cells cultured in differentiation medium containing human normal serum. All values are means ± standard deviation (*n* = 4). (**c**) The effects of muscle differentiation inhibition in the presence of DMSO or 0.1 μM AR-42 were evaluated as myotube area per field. Percentage of myotube area was quantified by normalizing to 0% for cells cultured in expansion medium as undifferentiated cells and 100% for cells cultured in differentiation medium containing human normal serum as differentiated cells. Muscle differentiation inhibition was induced by cancer patient sera (serum I, E, K, or L). All values are means ± standard deviations (*n* = 4). * *p* < 0.05 vs. DMSO (Holm–Sidak method). Abbreviations in the figure are as follows: DMSO, dimethyl sulfoxide. N.S., not significant.

Previous in vivo studies have reported the efficacy of the pan-HDAC inhibitor AR-42 in reducing muscle mass and fiber size in animal models of CC. Therefore, we tested its efficacy using the evaluation system constructed in this study [9]. Among the HDAC inhibitors evaluated to date, AR-42 demonstrated the most significant reducing effect at concentrations as low as 0.4 μM (Figure 6 and Figure 7a). At concentrations above 0.3 μM, the percentage of myotube area decreased, and at concentrations above 1 μM, the number of nuclei decreased (Figure 7a,b). Based on these results, we next tested the effect of AR-42 on various cancer patient sera at a concentration of 0.1 μM, where AR-42 exhibited the strongest reducing effect on muscle differentiation inhibition using four different cancer patient sera (I, E, K, L). Compared to the DMSO control, AR-42 approximately doubled the MHC-positive area percentage, confirming the reducing effects on muscle differentiation inhibition exerted by various cancer patient sera (Figure 7c).

## 3. Discussion

In vitro and in vivo cancer cachexia (CC) models are essential to accurately recapitulate the clinical features and pathophysiology of patients suffering from cachexia. A human cell-based model is especially needed because there is a great degree of heterogeneity in the transcriptomic and metabolic profiles when comparing human and rodent cells [39]. However, the complexity of CC, influenced by multiple factors, makes it challenging to reproduce in human models. Several pathophysiological stimuli that induce CC have been reported such as inflammatory factors [13], hormones [23], and cancer patient ascites [25]. Nevertheless, existing models might not fully capture this complexity of CC due to significant individual variability and the complex nature of the condition [40,41]. Several blood biomarkers of CC have been identified, including IL-6, TNFα, Angiotensin II, and GDF15 [42]. However, none have been universally adopted in clinical practice [42] and previous reports indicate that CC involves multiple factors in serum or plasma. Therefore, by using cancer patient sera, which has not been previously reported, we attempted to mimic the pathophysiological condition of CC in vitro.

Effects of CC on skeletal muscle include not only muscle atrophy but also impaired muscle regeneration. Muscle differentiation, which is a key step in skeletal muscle regeneration, was also impaired [43,44]. In our study, we confirmed the effects of TNFα, TWEAK, and cancer patient serum on muscle atrophy and the inhibition of muscle differentiation. Both effects were present in all stimuli, as shown in Figure 1, Figure 2 and Figure S1. These results suggest that CC may be intensified by the presence of both muscle atrophy and reduced muscle differentiation, which is necessary for muscle regeneration. Furthermore, the inhibitory effect of cancer patient sera on muscle differentiation was not reduced by the TNFα-neutralizing antibody (Figure 2f). The lack of efficacy of TNFα-neutralizing agents in clinical trials targeting cachexia, such as the monoclonal antibody infliximab [7] and the TNF receptor fusion protein etanercept [6], further supports the translational relevance of our screening system. Conversely, it has been reported that TNFα and TWEAK reduce the expression levels of key muscle differentiation factors during myogenesis [17,18,19,20]. In this study, the expression of key muscle differentiation factors such as *MyoD* and *MYOG* was also inhibited by cancer patient sera (Figure 2e). These results indicate that the reduction in muscle differentiation by cancer patient serum may be influenced by factors other than TNFα, and this assessment model reflects the pathophysiological state of the disease. To address the complexity of CC, we developed an in vitro screening system that used cancer patient serum as a pathophysiological stimulus for high-throughput screening (Figure 3).

It is important to confirm whether the screening system we have constructed, which mimics some key aspects of the pathophysiology of CC, correlates with the use of cancer patient sera and clinical data. The comparative evaluation of 12 different cancer patient sera (Table 1) using our 384-well plate-based screening system showed that serum from patients with cancers that show a high incidence of CC, such as pancreatic and colorectal cancer of grade III or higher, had a strong inhibitory effect on muscle differentiation (Figure 4). These results reflected the clinical characteristics of CC, at least in the sera studied [4]. This screening system uses 384 wells and is plate-based, and the future evaluation of more patient sera will allow us to assess the correlation with clinical data and serum biomarkers in multiple samples. The degree of effect on skeletal muscle can be evaluated from various patient sera. Thus, our system reflects the pathophysiology of CC and has significant potential for identifying multifactorial biomarkers and exploring the mechanisms of CC.

We identified active compounds using our screening system, revealing the possibility of finding new cachexia treatments through drug repositioning. Screening the FDA-approved drug compound library showed that only the proteasome inhibitor bortezomib produced a positive result (Figure 5). Bortezomib has been reported to inhibit muscle atrophy, and it might also influence muscle differentiation. One possible mechanism for the antagonism of muscle differentiation inhibition by bortezomib is the accumulation of the Rho protein due to the inhibition of its degradation [45], resulting in the promotion of muscle differentiation through Rho activation [46]. On the other hand, bortezomib has also been reported to cause muscle toxicity, posing challenges for its development as a treatment for cachexia from a safety perspective. To identify active compounds, we further screened an epigenetic compound focused library (Figure 5). Epigenetic changes contribute to the profound alteration in the transcriptional program associated with the onset and progression of muscle loss in several pathophysiological conditions [47]. Using our screening system, we identified several high-potential HDAC inhibitors including ITF2357 (Figure 6). The pan-HDAC inhibitor ITF2357 is approved as a treatment for Duchenne muscular dystrophy [48,49] and showed high activity in our system, indicating it may also be effective against cachexia-induced skeletal muscle loss. Our screening system identified that ITF2357 has activity in releasing muscle differentiation inhibition caused by cachexia, suggesting its potential effectiveness against cachexia-induced muscle loss. The most interesting datum is that AR-42 was most effective at the lowest concentrations and demonstrated the strongest reduction in differentiation inhibition (Figure 7a), whereas SAHA showed only partial activity (Figure 6). Previous reports have shown that the pan-HDAC inhibitor AR-42 significantly extended survival, while preventing the loss of muscle and preserving muscle fiber size [9]. However, SAHA did not show an anti-cachectic effect [9], aligning with our in vitro system’s limited effect of SAHA. Our screening system can obtain highly translational data that reflect the clinical data of TNFα-neutralizing antibodies and the in vivo cachexia model of HDAC inhibitors, making it a valuable tool for therapeutic drug screening.

Our phenotypic screening system pharmacologically analyzed the HDAC subtypes contributing to activity, revealing that a unique multiple inhibition profile targeting multiple HDAC subtypes is crucial for efficacy. In rodent cells, the effects of HDAC on muscle differentiation have already been reported, including HDAC1 regulating MyoD [50] and HDAC4, HDAC5, HDAC7, and HDAC9 regulating MEF2 [51,52]. The evaluation of 12 different HDAC inhibitors (Supplemental Table S1) using this phenotypic screening system showed high activity for the HDAC6-selective inhibitor CAY-10632, indicating that HDAC6 inhibition is a key contributor to activity. In contrast, the HDAC8-selective inhibitor PCI34051 showed no activity, suggesting that HDAC8 inhibition does not contribute to activity (Figure 6). The negative effects of HDAC6 on skeletal muscle have been reported [53], and the reversal of muscle differentiation inhibition by HDAC6 inhibitors is consistent with these findings. Pan-HDAC inhibitors showed high activity, but SAHA was an exception, showing only partial effects compared to other pan-HDAC inhibitors (Figure 6). 4-iodo-SAHA, which has a structure similar to SAHA, showed greater activity than SAHA, despite previous reports suggesting that both have comparable HDAC6 inhibitory activity [38]. This screening system is sensitive enough to detect even slight structural differences in activity, suggesting its potential utility in structure activity relationship (SAR) analysis. This indicates that the mechanism of action for reducing muscle differentiation inhibition cannot be explained solely by HDAC6 activity.

The broad efficacy of AR-42 across multiple patient sera indicates its potential as a widely effective treatment for cachexia (Figure 7c). This finding suggests that AR-42 could be beneficial across various patient profiles, regardless of the individual differences among cancer patients. Our screening system is expected to be used not only for the further optimization of AR-42 or SAHA but also for drug discovery.

The novel screening system we have constructed paves the way for innovative treatments for CC, facilitating the discovery of therapeutic agents, the identification of multifactorial biomarkers, and the exploration of mechanisms. Our screening system will not only assess the extent to which various patient sera affect skeletal muscle but will also allow us to narrow down the multifactorial influences on skeletal muscle by comparing blood biomarkers and patient symptoms. Drug screening is also possible, facilitating not only the optimization of AR-42 but also the development of compounds with different mechanisms. Cancer cachexia is a disease involving multiple organs, but since this evaluation system focuses on effects on skeletal muscle, it is essential to verify toxicity to other organs in vivo to comprehensively evaluate drug potential. Thus, our phenotypic screening system could be a pivotal tool for unraveling the complexity of cachexia and developing innovative therapies.

## 4. Materials and Methods

### 4.1. Human Cell Culture and Evaluation of Skeletal Muscle Differentiation and Atrophy

Human skeletal muscle myoblasts (HSMMs) were obtained from Lonza Walkersville, Inc. (Walkersville, MD, USA). HSMMs were utilized for this study. HSMMs were cultivated on collagen-coated dishes (Corning Incorporated, NY, USA, Cat #354450) in growth medium, SkBM-2 basal medium with supplements (Lonza, catalog #CC-2580), and incubated at 37 °C in a humidified atmosphere of 5% CO_2_ according to the manufacturer’s protocol and a previous report [26]. To induce myogenic differentiation, HSMMs were seeded in Matrigel-coated 384-well plates (Aurora Microplates, AZ, USA, Cat #ABM2-11101A) at a density of 2000 cells per well in growth medium using Micro Shot 705 (MS TECHNOS Co., Ltd., Tokyo, Japan). The plates were coated with Matrigel (Corning, NY, USA, cat#356234) before use. The cells were then incubated at 37 °C in a humidified atmosphere of 5% CO_2_. After 24 h of seeding, the medium was replaced with differentiation medium comprising DMEM/F12 (Gibco, MA, USA, cat #11320033), containing either 2% human normal serum or 2% cancer patient serum purchased from Tissue Solutions Ltd. (Glasgow, Scotland). To evaluate the effects that focused primarily on muscle differentiation, the cells were cultured and differentiated for four days, with the differentiation medium containing the test subjects. The endpoint was set at Day 4 of differentiation. To evaluate effects that focused primarily on atrophy, the cells were differentiated for five days in differentiation medium containing normal serum to mature the myotube adequately, and then cultured for four days in differentiation medium containing the test subjects. The endpoint was set at Day 9 of differentiation. Media were replaced every 48 h using EDR-384SR (BioTec Co. Ltd., Tokyo, Japan). In the TNFα neutralization test, anti-human TNFα neutralizing-antibody (R&D systems, Inc., USA, cat# AF-210-NA) was added to differentiation medium at a final concentration of 1 μg/mL [54,55] and preincubated for two hours, before initiating the differentiation process.

### 4.2. High Content, Phenotypic Screening

An epigenetic compound focused library, consisting of 95 compounds, was purchased from Cayman Chemical Company (Ann Arbor, MI, USA, cat #11076-0457556). We constructed the FDA-approved compound library, consisting of 712 compounds, using the Prestwich Chemical Library (Prestwick Chemical Inc., Illkirch-Graffenstaden, France) and SCREEN-WELL^®^ FDA-approved drug library V2 (ENZO Biochem, Inc., Farmingdale, NY, USA). At the initiation of differentiation, each compound was dissolved in DMEM/F12 containing 2% human cancer patient serum and the solution was added to each well of the assay plate at a final concentration of 1 μM or 10 μM using EDR-384SR. The cells were then incubated for four days to induce myogenic differentiation, with the medium replaced every 48 h, including the respective compound. For the analysis of myogenic differentiation, the cells were fixed with 4% paraformaldehyde and immune-stained with an antibody against fast myosin skeletal heavy chains (Abcam Limited, Cambridge, UK cat #51263). Data visualization was performed using TIBCO Spotfire (PerkinElmer, Inc., Waltham, MA, USA).

### 4.3. Immunostaining and Fluorescence Analysis

Cells were fixed with 4% paraformaldehyde (FUJIFILM Wako Pure Chemical Corporation, Osaka, Japan, cat #163-20145) for 20 min at room temperature (RT). After 2–3 rinses with PBS, cells were permeabilized with 0.5% TritonX-100 in PBS for 15 min at RT. Non-specific binding was blocked with 2% BSA and 0.25% Triton X-100 in PBS at RT for 30 min. Next, cells were incubated with anti-fast myosin skeletal heavy chain (Abcam, #ab512363), diluted 1:500 in blocking buffer for 2 h at RT. After the cells were rinsed with PBS three times, they were incubated with secondary antibody containing DAPI at RT for 1.5 h. Image acquisition was carried out with an InCell Analyzer 6000 (Cytiva, MA, USA). Data analysis was performed using InCell Investigator Developer Toolbox (1.9.2) software.

### 4.4. Quantitative Real Time PCR

Total RNA was extracted from cells using the Cells-to-CT Kit (Thermo Fisher Scientific Inc.) according to the manufacturer’s instructions. Briefly, cells were washed with phosphate-buffered saline (PBS) and lysed in the lysis buffer provided in the kit. The lysates were then subjected to reverse transcription to synthesize cDNA using the reverse transcription reagents included in the kit. Quantitative PCR (qPCR) was performed using TaqMan Gene Expression Assays (Thermo Fisher Scientific) to evaluate the expression levels of four muscle differentiation-related genes: troponin T type 1 (TNNT), myogenic differentiation factor 1 (MyoD), myocyte enhancer factor 2C (MEF2C), and myogenin (MYOG). The results are expressed as fold changes in mRNA expression normalized to beta-2 microglobulin (B2M) using the ∆∆Ct method.

### 4.5. Statistical Analysis

Statistical analyses and graph preparation were performed using Prism 6.0 (GraphPad Software Inc., San Diego, CA, USA). Data are presented as the mean, and each bar indicates the standard deviation. Dunnett’s multiple comparison method was used for comparing multiple serum treatments to a control group. The Holm–Sidak method was used for comparing the effects of compounds under each condition such as those treated with various sera. *p* values, where shown, indicate significance between each group (*, *p* < 0.05; **, *p* < 0.01; ***, *p* < 0.001; ****, *p* < 0.0001).

## 5. Conclusions

Our novel screening system effectively mimics the pathophysiology of some key aspects of cancer cachexia (CC) on skeletal muscle, providing a valuable tool for drug discovery and understanding the mechanisms of CC. Using cancer patient sera as pathophysiological stimuli, we demonstrated the significant inhibition of muscle differentiation, reflecting the clinical features of CC. We hypothesized that the anti-inflammation compounds within the FDA-approved library would be effective, given that sera from cancer patients contain multiple inflammatory cytokines and other pro-inflammatory substances. However, our system identified HDAC inhibitors as promising therapeutic agents, with a unique multiple inhibition profile targeting multiple HDAC subtypes being crucial for efficacy. The high translational relevance of our system, consistent with clinical and in vivo data, underscores its potential for identifying new treatments for and multifactorial biomarkers of CC. The high translational relevance of our system offers a promising avenue for therapeutic advancements in the management of CC.

## Figures and Tables

**Table 1:** Types of normal and cancer patient-derived human serum used, and corresponding information on patient diagnosis, cancer grading, and TNM classification.

¥	Patient Diagnosis	Grade	TNM Classification
A	– *	– *	– *
B	– *	– *	– *
C	Lung Cancer	III	T3, N0, M0
D	Lung Cancer	IIIA	T2, N2, M0
E	Colon Cancer	III	T3, Nx, M0
F	Colon Cancer	III	T3, N0, M0
G	Pancreatic Cancer	IV	Unknown
H	Pancreatic Cancer	IV	Unknown
I	Stomach Cancer	IV	T3, Nx, M1
J	Stomach Cancer	Unknown	Unknown
K	Pancreatic Cancer	III	T4, N0, M0
L	Pancreatic Cancer	III	T4, N0, M0
M	Pancreatic Cancer	IV	Unknown

* Sera A and B comprise human normal serum, which do not have diagnostic data. Grade: Description of a tumor based on how abnormal the cancer cells and tissue appear under the microscope. Higher-grade cancers tend to grow and spread more rapidly than lower-grade cancers. TMN classification: cancer categorization based on three components: the size and extent of the primary tumor (T), the involvement of regional lymph nodes (*n*), and the presence of distant metastasis (M).

## Data Availability

The data presented in this study are contained within the article and Supplementary Materials. Requests for further details can be directed to the corresponding author.

## Supplementary Materials

The following supporting information can be downloaded at: https://www.mdpi.com/article/10.3390/ph18040445/s1, Figure S1: Effects of cancer patient serum on myotubes; Table S1: Reference data for IC_50_ or %inhibition values of HDAC inhibitors.
