# Supplementary material for: Advanced High-Content Phenotypic Screening to Identify Drugs That Ameliorate the Inhibition of Skeletal Muscle Cell Differentiation Induced by Cancer Cachexia Serum
Source: Pharmaceuticals (Basel). 2025 Mar 21;18(4):445. doi: 10.3390/ph18040445 (PMC12030060; doi:10.3390/ph18040445)
Supplement: Supplementary file 1 [file pharmaceuticals-18-00445-s001.zip › Figure S1.pdf]

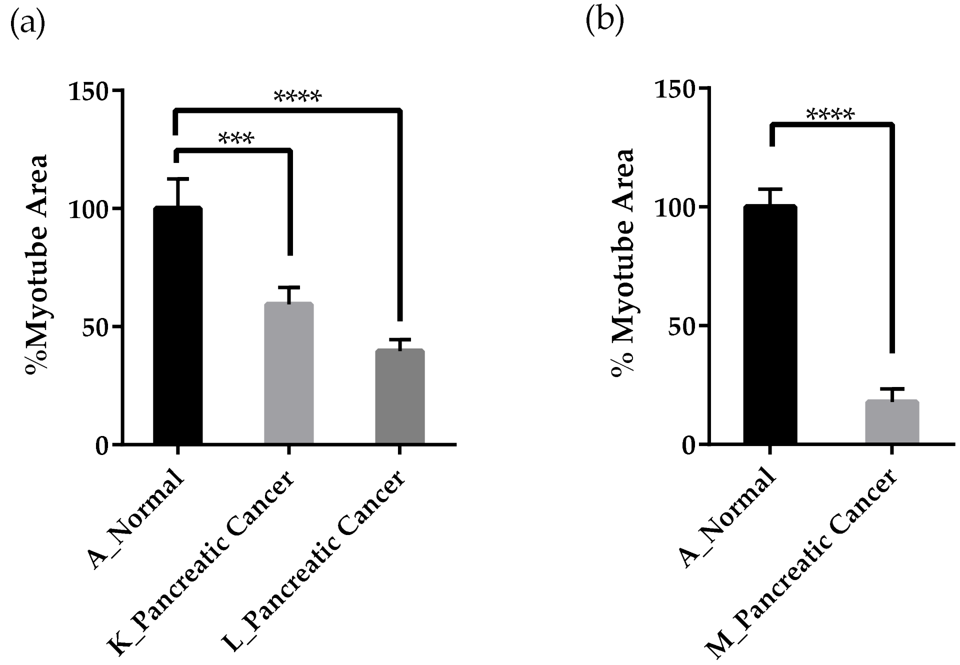

Figure S1 Effects of various types of cancer patient serum on myotube area.
Quantification of % myotube area per field on Day 4 in differentiation medium containing normal serum (A) or cancer patient sera K, L, or M). All values are means ± standard deviations (n = 4). Percentage myotube area per field is quantified by normalizing to 0% for cells cultured in expansion medium as undifferentiated cells and 100% for cells cultured in differentiation medium containing normal human serum *** Denotes a significant difference from normal serum A at $p < 0.001$, and **** denotes a significant difference at $p < 0.0001$ (Dunnett's multiple comparisons test for (a) or Student T test for (b)).
