# Supplementary material for: Advanced High-Content Phenotypic Screening to Identify Drugs That Ameliorate the Inhibition of Skeletal Muscle Cell Differentiation Induced by Cancer Cachexia Serum
Source: Pharmaceuticals (Basel). 2025 Mar 21;18(4):445. doi: 10.3390/ph18040445 (PMC12030060; doi:10.3390/ph18040445)
Supplement: Supplementary file 1 [file pharmaceuticals-18-00445-s001.zip › Table S1.pdf]

	Reference data for IC_{50} or %inhibition values of HDAC inhibitors (μM)											
	Class Ⅰ HDAC				Class Ⅱa HDAC				Class Ⅱb HDAC		ClassⅣ HDAC	
**Compound**	**1**	**2**	**3**	**8**	**4**	**5**	**7**	**9**	**6**	**10**	**11**	**Ref.**
SB939	0.049	0.096	0.043	0.14	0.056	0.047	0.137	0.07	1.008	0.04	-	[31]
Chidamide	0.10	0.2	0.1	0.7	>10	>10	>10	>10	>10	0.1	0.4	[32]
MS275	0.2	1.2	2.3	>10	>10	-	>10	0.5	>10	-	-	[34]
SAHA	0.06	0.042	0.036	0.173	0.02	0.036	0.129	0.049	0.029	0.06	0.031	[32]
ITF2357	0.13	0.29	0.14	0.84	>1.00	0.53	0.52	0.51	0.31	0.33	0.29	[32]
KD5170	0.02	2.06	0.08	2.50	0.03	0.95	0.09	0.15	0.01	0.02	-	[33]
TSA	0.01	0.02	0.01	0.12	0.02	0.02	0.08	0.08	0.001	0.03	-	[33]
CAY-10603	0.27	0.25	0.00042	6.85	-.	-	-	-	0.000002	0.09	-	[34]
TC-H 106	0.15	0.76	0.37	5	>180	>180	>180	-	-	-	-	[35]
PCI34051	4	>50	>50	0.01	-	-	-	-	2.90	13.00	-	[34]
AR-42	*95%	*72%	*93%	*87%	*21%	*0%	*22%	*41%	*100%	*86%	-	[37]

N.D. not determined, Symbol: -, No available information in the literatures. * Data of AR-42 means % inhibition at 1μM.
